# The Influence of Gd-EOB-DTPA on T2 Signal Behavior: An Example from Clinical Routine

**DOI:** 10.3390/diagnostics12081811

**Published:** 2022-07-28

**Authors:** Paola Franceschi, Anna Pecorelli, Rita Golfieri, Matteo Renzulli

**Affiliations:** Department of Radiology, IRCSS Azienda Ospedaliero Universitaria di Bologna, 40138 Bologna, Italy; paola.franceschi@studio.unibo.it (P.F.); anna.pecorelli@aosp.bo.it (A.P.); rita.golfieri@unibo.it (R.G.)

**Keywords:** magnetic resonance imaging (MRI), hepatic hemangioma, hepatospecific contrast agent, Gd-EOB-DTPA

## Abstract

In the literature, it has repeatedly been stated that the introduction of hepatospecific contrast agents in Magnetic Resonance Imaging prolongs the acquisition time due to the hepatobiliary phase, normally acquired 15–20 min after injection. Many efforts have been made to shorten the time-consuming protocols, and it was demonstrated that T2-Weighted Images (T2WI) and Diffusion-Weighted Images (DWI) acquired after Gd-EOB-DTPA show a comparable diagnostic capability to pre-contrast T2WI and DWI in the detection and characterization of hepatic tumors. Therefore, T2WI and DWI are usually acquired after the acquisition of vascular phases, in the dead time until the acquisition of the hepatobiliary phase. Unfortunately, contrast agents, especially Gd-EOB-DTPA, reduce the hydrogen nuclei’s relaxation time and modify signal intensity. We report a case in which, due to these limitations of the acquisition protocol, two hemangiomas showed an inhomogeneous, low signal on T2WI and DWI that was not visible in a follow-up scan a few days later. In conclusion, when liver lesions of unknown nature must be characterized, and there is a lack of previous radiological investigations, it could be useful to acquire pre-contrast T2WI and DWI to avoid diagnostic confusion, especially in non-tertiary centers.

**Figure 1:**
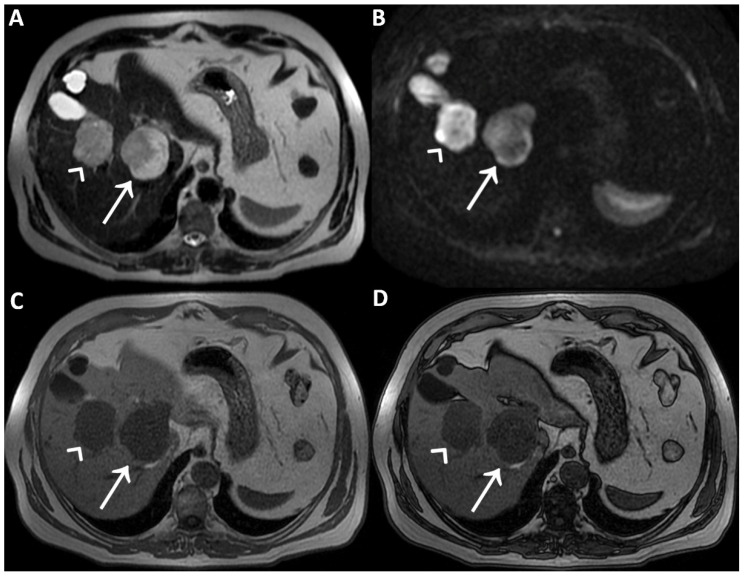
A man in his early 60s with unremarkable medical history and normal laboratory data underwent an abdominal ultrasound for insurance reasons, which detected two liver lesions, the largest one localized deeply in the hepatic parenchyma. Due to the patient’s high body mass index, which reduced the ultrasound’s diagnostic quality, panoramic imaging was required to achieve the final diagnosis. Therefore, the patient underwent liver Magnetic Resonance Imaging (MRI) performed with hepatospecific contrast agent (Gd-EOB-DTPA). MRI was performed by using a 1.5 T superconducting system (Signa; GE Medical Systems, Milwaukee, Brookfield, WI, USA) with a body phased-array multicoil for signal detection. The two liver lesions appeared to be inhomogeneously hyperintense on T2-Weighted Images (T2WI) (straight arrow and arrowhead in (**A**)) and on Diffusion-Weighted Images with b-value = 800 (DWI) (straight arrow and arrowhead in (**B**)) and homogeneously hypointense on T1-Weighted Images (T1WI) (straight arrow and arrowhead in (**C**), in-phase T1WI, and (**D**) opposed-phase T1WI). It was not possible to establish the benignity of the two liver lesions evaluating T2WI and DWI acquired after contrast agent administration, in particular due to the inhomogeneous signal intensity shown on T2WI. To ascertain that it had not been caused by the contrast-induced reduction in the hydrogen nuclei relaxation time, it was necessary to re-acquire the same images without previous contrast administration a few days later.

**Figure 2:**
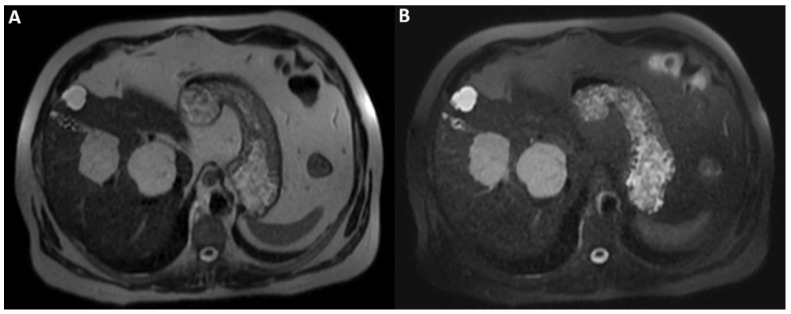
On T2WI without fat-suppression (**A**) and with fat-suppression (**B**) acquired a few days later without previous contrast agent administration; the lesions’ hyperintensity was clearly homogeneous. Therefore, the inhomogeneous signal intensity of the lesions on post-contrast T2WI is a pseudo-inhomogeneity caused by limitations of the acquisition protocol and a direct consequence of the contrast-induced reduction in the hydrogen nuclei relaxation time. Consequently, it was possible to establish the lesions’ benignity and make the final diagnosis of cavernous hemangiomas.

**Figure 3:**
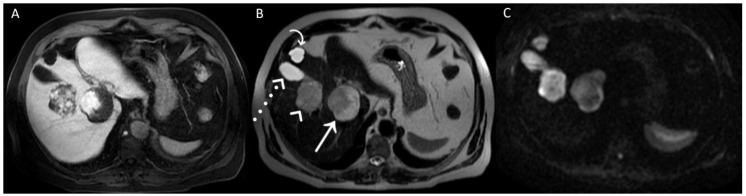
A posteriori, a perfect correspondence between the contrast agent globular enhancement on Axial T1-weighted fat saturated images in delayed phase (**A**) and the relatively hypointense areas on post-contrast T2WI (**B**) and DWI (**C**) was evident. Furthermore, the largest and deepest lesion showed a relatively hypointense area on DWI, larger than that on T2WI, due to later acquisition of DWI and progressive centripetal contrast filling of the lesion. On T2WI (**B**), we can also appreciate the comparison between the signal hyperintensity of different structures: cavernous hemangiomas (arrow and arrowhead); simple cyst (curved arrow), and gallbladder’s fundus (dotted arrow). T2WI and DWI are usually acquired after the acquisition of vascular phases in the dead time until the acquisition of the hepatobiliary phase to shorten the time-consuming protocols since it was demonstrated that T2WI and DWI acquired after Gd-EOB-DTPA showed a comparable diagnostic capability to pre-contrast T2WI and DWI in the detection and characterization of hepatic tumors [1,2]. In conclusion, when liver lesions of unknown nature must be characterized, with a lack of previous radiological investigations, it could be useful to acquire pre-contrast T2WI and DWI to avoid diagnostic confusion, especially in non-tertiary centers.

**Figure 4:**
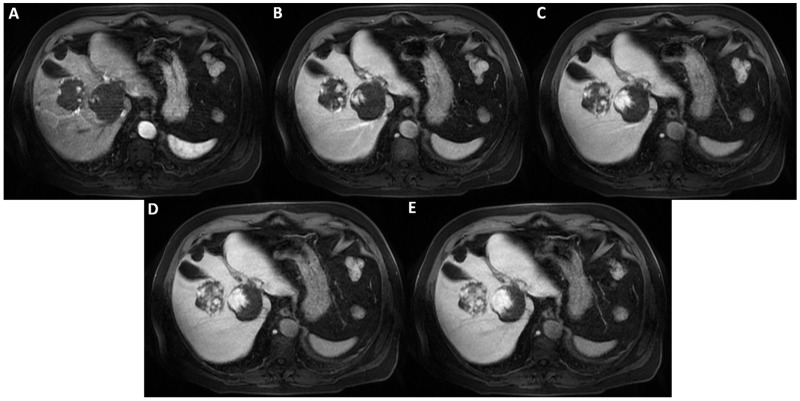
Axial T1-weighted fat saturated images after hepatospecific contrast agent (Gd-EOB-DTPA) administration in arterial, portal-venous, and delayed phases (**A**–**E**) show early peripheral discontinuous globular enhancement of the lesions with progressive centripetal filling-in on delayed scans. This angiodynamic behavior supports the diagnosis of cavernous hemangiomas.

## Data Availability

Not applicable.
